# Cretaceous basin evolution in northeast Asia: tectonic responses to the paleo-Pacific plate subduction

**DOI:** 10.1093/nsr/nwab088

**Published:** 2021-05-18

**Authors:** Qing-Ren Meng, Zhong-He Zhou, Ri-Xiang Zhu, Yi-Gang Xu, Zheng-Tang Guo

**Affiliations:** State Key Laboratory of Lithospheric Evolution, Institute of Geology and Geophysics, Chinese Academy of Sciences, Beijing 100029, China; Innovation Academy for Earth Science, Chinese Academy of Sciences, Beijing 100029, China; College of Earth and Planetary Sciences, University of Chinese Academy of Sciences, Beijing 100049, China; College of Earth and Planetary Sciences, University of Chinese Academy of Sciences, Beijing 100049, China; Key Laboratory of Vertebrate Evolution and Human Origins, Institute of Vertebrate Paleontology and Paleoanthropology, Chinese Academy of Sciences, Beijing 100044, China; State Key Laboratory of Lithospheric Evolution, Institute of Geology and Geophysics, Chinese Academy of Sciences, Beijing 100029, China; Innovation Academy for Earth Science, Chinese Academy of Sciences, Beijing 100029, China; College of Earth and Planetary Sciences, University of Chinese Academy of Sciences, Beijing 100049, China; Innovation Academy for Earth Science, Chinese Academy of Sciences, Beijing 100029, China; College of Earth and Planetary Sciences, University of Chinese Academy of Sciences, Beijing 100049, China; State Key Laboratory of Isotope Geochemistry, Guangzhou Institute of Geochemistry, Chinese Academy of Sciences, Guangzhou 510640, China; Innovation Academy for Earth Science, Chinese Academy of Sciences, Beijing 100029, China; College of Earth and Planetary Sciences, University of Chinese Academy of Sciences, Beijing 100049, China; Key Laboratory of Cenozoic Geology and Environment, Chinese Academy of Sciences, Beijing 100029, China

**Keywords:** NE Asia, Cretaceous, rift basin, volcanism, paleo-Pacific plate

## Abstract

Cretaceous rift basin evolution was an important part of the tectonic history of northeast Asia in the late Mesozoic. Three types of rift basins are identified—active, passive and wide rift basins—and they developed in different regions. Passive rift basins in the eastern North China craton are thought to be the consequence of crustal stretching and passive asthenospheric upwelling. Wide rift basins in the eastern Central Asian orogen are assumed to originate from gravitational collapse of the thickened and heated orogenic crust. Active rift basins in the northern North China craton are attributed to uprising of asthenospheric materials along a lithospheric-scale tear fault. Slab tearing of the subducting paleo-Pacific plate is postulated and well explains the spatial distribution of different types of rift basins and the eastward shifting of magmatism in the northern North China craton. The Late Cretaceous witnessed a period of mild deformation and weak magmatism, which was possibly due to kinematic variation of the paleo-Pacific plate.

## Teaser

Cretaceous basins in NE Asia originated from three types of continental rifting in disparate regions and evolved in response to differential subduction and slab tearing of the western paleo-Pacific plate.

## INTRODUCTION

**Figure 1.**
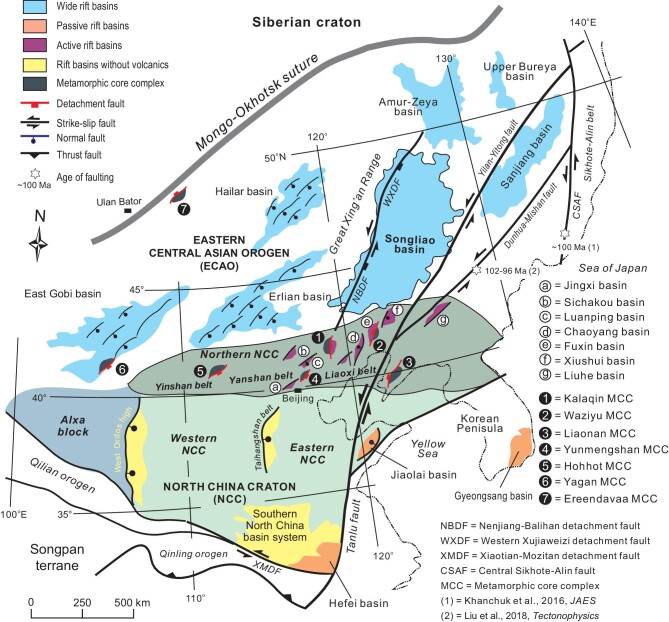
Tectonic map showing distribution of Cretaceous basins in NE Asia. The NE Asian continent is divided into two main domains, the North China craton (NCC) and eastern Central Asian orogen (ECAO). The NCC can be further divided into three parts, the western, eastern and northern NCC, based on their distinct tectonic evolution in late Mesozoic. Note that passive, active and wide rift basins are distributed in different regions, with metamorphic core complexes being closely associated with active rift basins.

The northeastern Asian continent experienced alternating crustal contraction and extension as well as sporadic magmatism in the Mesozoic [1–4]. The multiple tectono-magmatic processes are ascribed to the near-field and far-field effects of the changes in subduction angles of the paleo-Pacific plate [5,6], continent–continent collisions and the resulting escape tectonics [7–9], subcontinental thermo-tectonic processes [10–12], and a combination of diverse tectonic drivers [13]. Two phases of strong crustal shortening, which took place in the late Middle Jurassic and at the end of the Late Jurassic [2,14,15], have been identified and extensively studied. The end-Jurassic contraction was intense and extensive, as indicated by widespread folding and thrusting as well as a regional angular unconformity beneath Lower Cretaceous strata [2,16]. This phase of shortening, termed Phase B of the Yanshanian orogeny in the literature [17], resulted in two main consequences: crustal thickening of the eastern Central Asian orogen (ECAO) and the onset of destabilization of the North China craton (NCC). Extensive rifting occurred in the aftermath of this contractional event [18,19]. Early Cretaceous rift basins developed throughout the northeastern Asian continent and expressed themselves in general as disparate small- and mediate-scale basins (Fig. 1). Vigorous volcanism accompanied the rifting [3,12,20], with volcanic/volcaniclastic rocks making up significant parts of most basin successions. Previous studies focused mainly on individual rift basins in different regions, such as the Erlian, Hailar and Songliao basins in the ECAO [21–23], the Luanping basin in the northern NCC [24] and the Hefei and Jiaolai basins in the eastern NCC [25].

Distinct rift basins are distributed in different regions, as hinted by diverse Lower Cretaceous volcano-sedimentary sequences. The basins in the eastern NCC started with clastic sedimentation, which was followed by volcanic eruption [25,26]. By contrast, volcanism marked the initiation of the rift basins in the northern NCC, and clastic deposition then succeeded [24]. Volcaniclastic and volcanic rocks are present throughout basin sequences in the ECAO [21,23]. The existing tectonic models, however, seldom explicate how the diverse rift basins are generated simultaneously and why they are distributed in different areas. Early Cretaceous rifting in NE Asia is commonly attributed to backarc extension induced by the westward subduction of the paleo-Pacific plate [1,25]. Unfortunately, it remains poorly known why extension basically came to an end during the Late Cretaceous and what caused differential basin subsidence in space. This study takes a holistic treatment of tectonic evolution of Cretaceous rift basins in NE Asia and attempts to explore the dynamic controls of time-space variations of the rift basins.

## TECTONIC SETTING

The northeast Asian continent is made up of two tectonic domains, the NCC in the south and the ECAO in the north (Fig. 1). The NCC developed as a single stable tectonic domain from the Mesoproterozoic to Paleozoic, and underwent little crustal deformation and magmatism for over 1.20 Ga [1,3]. The NCC kept its stability as a whole in the early Mesozoic albeit its peripheral regions were affected by terrane accretion as a result of the closure of the paleo-Asian and paleo-Tethyan oceans [27]. The late Mesozoic was a period when the different portions of the NCC began experiencing diverse thermo-tectonic evolution. The western NCC still behaved as a stable element with few tectonic activities. In contrast, the eastern NCC was characterized by lithospheric thinning and extensive magmatism, and completely lost its stability by the Early Cretaceous [1,3,11]. The northern NCC manifests itself as a unique zone by virtue of strong extension and magmatic outpouring, which were interrupted by short-term crustal/lithospheric shortening [1,2]. Compared with the NCC, the ECAO was built up with a number of terranes that had been amalgamated by the end of the Paleozoic [28]. The ECAO is therefore a wide orogenic domain with complex crustal compositions and fabrics.

**Figure 2.**
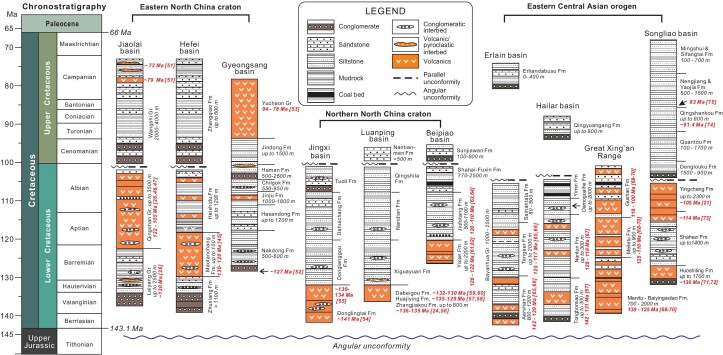
Stratigraphic correlation of Cretaceous sequences in NE Asia. Lower Cretaceous strata in the eastern NCC start with clastic rocks, which are followed by volcanic and volcaniclastic rocks. In contrast, Lower Cretaceous sequences in the northern NCC are marked by volcanic and volcaniclastic rocks in the lower part and clastics in the upper. In the ECAO, clastic rocks dominate Lower Cretaceous successions in the western portion, whereas volcanic and volcaniclastic rocks are prevalent in the eastern portion. The Songliao basin possesses thick Upper Cretaceous strata in contrast with other basins. The number in square brackets refers to numbering of the reference list.

Cretaceous extensional tectonics in NE Asia are evidenced by rift basins, metamorphic core complexes and vigorous magmatism [2,24,29–33]. In addition, the NCC lithosphere was significantly attenuated and experienced a radical change from continental to oceanic lithospheric mantle [34,35]. These tectonic processes happened mainly in the Early Cretaceous and led to total destabilization of the eastern NCC [36]. Early Cretaceous extension and magmatism in the eastern NCC were in essence the surface expressions of deep thermo-mechanic processes, which were possibly associated with a big mantle wedge system resulting from rollback and retreat of the subducting paleo-Pacific plate [1,10]. Coeval extension in the ECAO is thought to be the consequence of gravitational collapse of the thickened orogenic crust [29,37]. Crustal thickening might have resulted partly from the collision of the ECAO and Siberian craton along the Mongo-Okhotsk suture [38] and partly from the tectonic push due to flat subduction of the paleo-Pacific plate at the Jurassic to Cretaceous transition [1,39]. This shortening event is registered by a regional unconformity beneath the Lower Cretaceous in the ECAO [9] (Fig. 2). Late Cretaceous tectonics of NE Asia was characterized by vertical crustal motion, with Early Cretaceous rift basins either undergoing uplift/erosion or subsidence. The small-scale basins in both the ECAO and the northern NCC were uplifted or inversed at the end of Early Cretaceous, with a few Upper Cretaceous strata left (Fig. 2). The Songliao basin, situated in the east of the ECAO, is an exception in that it experienced pronounced sagging during the Late Cretaceous [21]. The Jiaolai basins in the eastern NCC also underwent striking subsidence in the Late Cretaceous, which is assumed to have had a bearing on the strike-slip motion of the Tanlu fault [26]. Large-scale sinistral transpression happened ∼100 Ma along the eastern margin of the NE Asian continent, as manifested by the occurrence/reactivation of left-slip faulting, such as the Tanlu fault [5], Dunhua-Mishan fault [40] and the Central Sikhote-Alin fault [41].

## BASIN SEQUENCES

Cretaceous strata are well preserved in NE Asia, and both lithostratigraphic and biostratigraphic sequences have been intensively investigated. Basins in different regions display distinct volcano-sedimentary sequences (Fig. 2). The ages of lithostratigraphic units are tightly constrained by precise U-Pb zircon and Ar-Ar dating of volcanic and volcaniclastic beds in conjunction with fossil assemblages. Cretaceous successions are separated from the underlying units by a regional angular unconformity, which registers a strong shortening event just prior to the Early Cretaceous rifting. Another unconformity occurs between the Lower and Upper Cretaceous, and manifests itself as either a parallel or low-angle discordant surface (Fig. 2). Cretaceous strata are unconformably covered by or pass upward conformably to Tertiary sediments [25,42].

Complete Cretaceous successions in the eastern NCC are best preserved in the Jiaolai and Hefei basins (Fig. 2). Lower Cretaceous succession displays two distinct parts, with the lower dominated by clastic rocks and the upper by volcanic and volcaniclastic rocks. The clastic parts are represented by the Laiyang Group in the Jiaolai basin and by the Zhuxiang Formation in the Hefei basin. Fluvial conglomerate and sandstone facies associations make up the lower part of the Laiyang Group, whereas the upper part consists primarily of meandering fluvial and lacustrine facies [43]. The Shuinan Formation, a unit in the middle Laiyang Group, contains a basalt layer that yields an ^40^Ar/^39^Ar plateau age of 129.7 ± 1.7 Ma [26]. The Zhuxiang/Fenghuangtai Formation in the Hefei basin shares similar facies to the lower Laiyang Group, and has an accumulative thickness up to 2500 m [44,45]. Clastic sedimentation was suppressed by vigorous volcanism, as indicated by a rapid change from siliciclastic to volcaniclastic and/or volcanic rocks that dominate the upper parts of the Lower Cretaceous successions of both the Hefei and Jiaolai basins (Fig. 2). The Maotanchang volcanics, up to 1000 m thick, represent late volcanism in the Hefei basin, and range in age from 130 to 120 Ma [45]. The Maotanchang volcanics pass upward into the Heishidu Formation, which is dominated by lacustrine fine-grained facies and contains abundant pyroclastic rocks [44]. The Qingshan Group, up to 1500 m thick, comprises basic and felsic volcanic rocks that yield ^40^Ar/^39^Ar plateau ages from 122 to 105 Ma [26,46,47]. Lower Cretaceous sequences are overlain unconformably by Upper Cretaceous strata, such as the Wangshi Group in the Jiaolai basin and the Zhangqiao Formation in the Hefei basin (Fig. 2). The Wangshi Group consists mostly of alluvial–fluvial coarse-grained facies, with depositional ages ranging from 107 to 73.5 Ma based on radiometric ages of volcanic beds and detrital zircons [48,49]. An angular unconformity separates the Lower from Upper Cretaceous units in the eastern NCC [26]. The discordant contacts are both observed at outcrops and identified on seismic profiles that are near or within the Tanlu fault zone [26,50].

Cretaceous sequences commence with volcanics in the northern NCC, as recorded by the Donglingtai Formation in the western segment, the Zhangjiakou Formation in the middle segment and the Yixian Formation in the eastern segment (Fig. 2). The Zhangjiakou and Donglingtai volcanics in the western Yanshan belt are dated at 143 ± 0.67 Ma, 143.4 ± 0.65 Ma and 140.7 ± 0.64 Ma [54,76,77], whereas the Zhangjiakou rhyolite and ignimbrite in the eastern Yanshan belt yield U-Pb zircon ages ranging from 136 to 131 Ma [24,56,60]. The Yixian volcanics have both ^40^Ar/^39^Ar plateau ages and U-Pb zircon ages ranging from 126 to 124 Ma [61,78]. Accordingly, Early Cretaceous volcanism became younger eastward in the northern NCC [10,11]. Clastic sedimentation then took the place of volcanism with time, and prevailed in the late stage, as recorded by the Xiguayuan and Jiufotang Formations in different basins (Fig. 2). The clastic units are collectively assigned to the Hauterivian to Aptian ages based on radiometric ages and fossil assemblages [64], and are characterized by fluvial-lacustrine facies associations [24,79]. The Qingshila, Shahai and Fuxin Formations represent the uppermost portions of Lower Cretaceous successions, and are made up mostly of fluvial facies associations. The Upper Cretaceous, if present, is separated from older units by either disconformities or low-angle unconformities (Fig. 2).

Lower Cretaceous strata are extensively preserved in the ECAO, and composed primarily of clastic facies [29,37,80]. Volcanics, usually present as interlayers, also occur in Lower Cretaceous successions of the East Gobi, Erlian and Hailar basins, and are largely basalt and basaltic andesite, yielding ^40^Ar/^39^Ar ages from 142 to 113 Ma [22,29,65,66,80–82]. Early Cretaceous volcanics and volcaniclastic rocks are widespread in the Great Xing’an Range, and dated at 135–115 Ma [20,68,69,81]. The Lower Cretaceous succession of the Songliao basin contains thick volcanic and volcaniclastic rocks, such as the Huoshiling and Yingcheng Formations, albeit clastic facies are also commonplace (Fig. 2). The Huoshiling volcanics are recently dated at 133–129 Ma [71,72], much younger than the previous age assignment of ∼150 Ma [21]. The Yingcheng volcanics are constrained at 120–105 Ma [21], indicating the persistence of volcanism to the end of the Early Cretaceous. The Upper Cretaceous is well developed in the Songliao basin, up to 3 km thick [83]. In contrast, the rift basins in the western portion of the ECAO possess meager Upper Cretaceous strata, which are usually less than 500 m thick [29]. A regional unconformity separates the Lower from Upper Cretaceous [21,29].

## MAGMATISM

Vigorous volcanism and plutonism characterized the NE Asian continent during the Early Cretaceous [3,20,32]. Volcanic rocks are widely distributed in the northern NCC, as represented by the Donglingtai andesite in the West Hill, Zhangjiakou rhyolite in northern Hebei and Yixian basalts in western Liaoning. Volcanism in the eastern NCC took place in the late stage of rift basin development, as recorded by the Qingshan rhyolite and basalt in the Jiaolai basin, the Maotanchang andesite in the Hefei basin, the Laohutai basalts in the Fushun basin [84] and Xiaoling Formation in eastern Liaoning [85]. Early Cretaceous volcanism was vigorous in the Great Xing’an Range and the Songliao basin [20,21,70,81], but declined significantly westward. Volcanic and/or volcaniclastic rocks are only present as interlayers in the lower successions of the East Gobi [80] and Erlian basins [66].

Early Cretaceous intrusions are also extensive in NE Asia [3,32]. Mafic intrusives occur in the eastern NCC, such as gabbro-pyroxenite complexes in the Taihang Shan belt and diorite/gabbro bodies in western Shandong [86,87]. Granitoids are distributed in the periphery of the eastern NCC [3,32], such as the Fangshan granite in the western Yanshan belt [88], the Sanguliu granite in the eastern Liaoning belt [11] in the northern NCC, the Guojialing granite in the Jiaodong Peninsula in the NCC eastern margin [89] and the Huashan and Heyu granites at the southern edge of the NCC [90]. Early Cretaceous granitoids are also widely documented in the ECAO, particularly in NE China [32,91].

**Figure 3.**
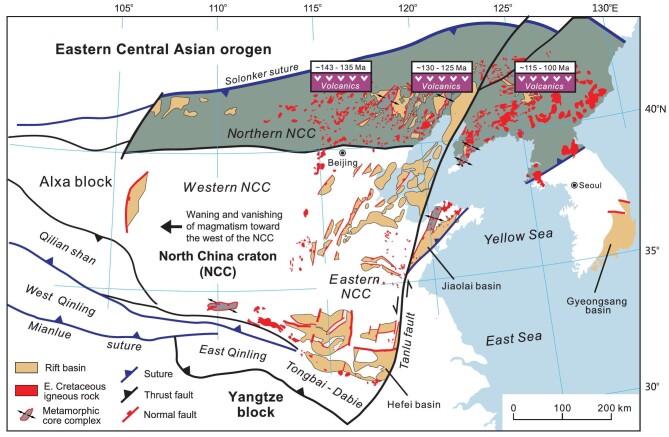
Distribution of Early Cretaceous igneous rocks in the NCC. Note that igneous rocks are particularly abundant in the northern NCC and both intrusive and extrusive rocks display a marked east-younging trend.

Growing geochronologic data show that Early Cretaceous magmatism took place during a wide range of time although it mainly happened from 130 to 120 Ma [32]. An east-younging trend in magmatic activity has been recognized [10,11], and is best demonstrated by eastward progression of Early Cretaceous volcanism in the northern NCC (Fig. 3). Volcanism started at 143–140 Ma in the Yinshan and western Yanshan belts [3,54,77], ∼136 Ma in the eastern Yanshan belt [56], ∼126 Ma in the Liaoxi region and ∼120–110 Ma in the Liaodong region [32,85]. Early Cretaceous magmatism in the eastern NCC commenced around 138 Ma in the Taihangshan belt [92], and appeared to have not started until ∼130 Ma in the easternmost NCC [87]. No matter when it began, Early Cretaceous magmatism lasted until ∼110 Ma within and around the eastern NCC [11,32]. Early Cretaceous magmatism in the ECAO occurred from ∼130 to 110 Ma, and was vigorous in the east [70,81].

Also noticeable is the time-space variation of magma types in the eastern NCC. Felsic and intermediate magmatism was prevailing in the west from 143 to 136 Ma, whereas mafic magmatism took place largely in the east from 130 to 110 Ma [87]. The eastward migration of magmatism was also associated with an increase in alkaline and mafic rocks like syenite and gabbro [93]. This situation is well exemplified by ∼143 Ma rhyolites and granitoids in the western Yanshan and Taihangshan belts [3,54] and ∼130–110 Ma mafic rocks in the easternmost NCC [87]. Magmatism persisted in the western portion of the eastern NCC when migrating eastwards [11]. Felsic volcano-plutonic associations are also common in the easternmost NCC, coeval with mafic and alkaline magmatism [32,87].

Early Cretaceous granite in the northern NCC is shown to have formed at high temperature ranging from 640 to 1100^o^C with a peak at ∼770^o^C [11]. This deduction is supported by the co-occurrence of mafic rocks that originate from high-temperature melts [87]. Coexistence of felsic and mafic magmas in the northern NCC indicate intense crust–mantle interaction [94], thereby hinting at the uprising of hot asthenospheric materials.

Magmatism declined significantly throughout the NCC and ECAO in the Late Cretaceous [3,4], and occurred largely along the eastern edge of the NE Asian continent, such as the Sikhote Alin belt [95], Korea [96] and southwestern Japan [91]. Late Cretaceous igneous rocks are mostly granitoid, andesite and pyroclastic rocks, representing island-arc magmatic activities triggered by the paleo-Pacific plate subduction [69,97].

## BASIN EVOLUTION

Rift basins are usually classified on the basis of dynamic, geometric and kinematic aspects, such as active and passive rifts [98] and wide and narrow rifts [99,100]. Active rifting is attributed to active uprising of mantle plumes, which first leads to doming and then induces supracrustal stretching [101]. Passive rifting is ascribed to lithosphere extension as a result of horizontal in-plane far-field forces, with the asthenospheric materials rising passively due to lithosphere thinning [101]. Wide rift systems develop owing to gravitational collapse of the orogenically thickened crust. Tensile deviatoric stress fields in the thickened crust are produced by lateral variation in gravitational potential energy [102]. By contrast, narrow rifts result from necking of the lithosphere with normal geotherm and crust thickness [100], and therefore fit the passive rifting mode. Merle (2010) proposes a rift classification in the context of tectonic settings, such as subduction-, mantle-, transform- and mountain-related rifts [103]. However, it is a purely interpretive classification and cannot help explore the real mechanism of continental rifting. Moreover, wide rifts are neither taken into account in Merle's classification nor readily fall into the category of Sengör and Burke's (1978) classification [98]. Active and passive rifting modes do have drawbacks and cannot successfully explain the whole evolution of continental rifts [103,104]. However, this simple classification proves quite useful for the first-order assessment of continental rifting [105]. Obviously, no existing rift classifications can encompass all types of continental rifts and no single driving force can account for all aspects of rift basins. We thus take a pragmatic approach to dealing with Cretaceous rift basins in NE Asia by adopting the categorization of active, passive and wide rifting. Our rationale is that the investigated basin successions appear to be compatible with the distinct rifting modes. The three types of rifting are thus considered to originate from three driving forces: (i) far-field forces originating at plate boundaries; (ii) forces acting on the base of the lithosphere due to the asthenospheric uprising, and (iii) buoyancy forces arising within the thickened orogenic crust. Different drivers may work together to control the development of some rift basins.

**Figure 4.**
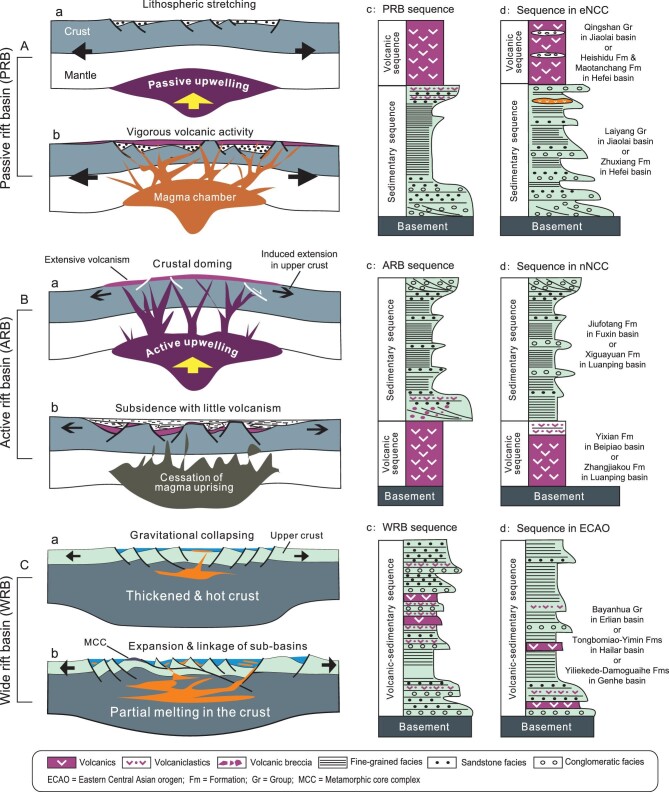
Models for tectonic subsidence of different types of rift basins and resultant volcano-sedimentary sequences. (A) Passive rift basin. (B) Active rift basin. (C) Wide rift basin. Refer to text for detailed explanation. eNCC = eastern NCC; nNCC = northern NCC.

**Figure 5.**
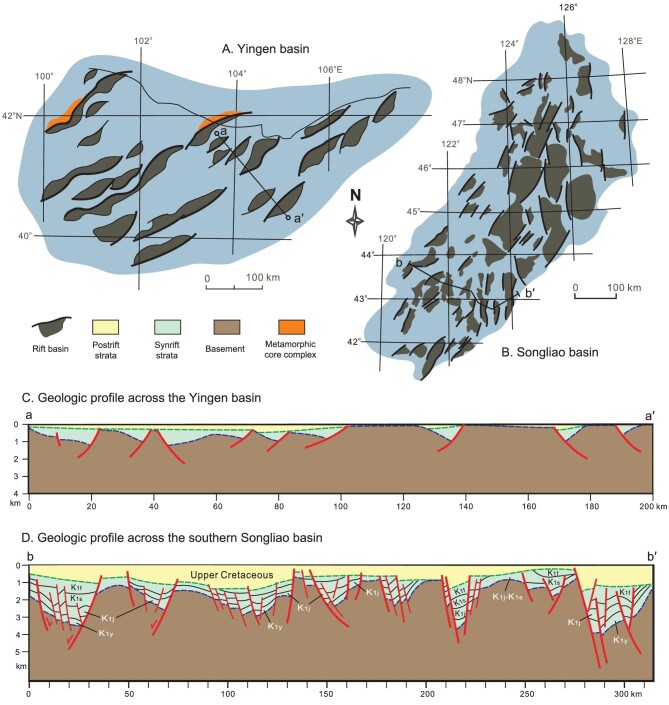
Diagrams showing structures of rift basins in the ECAO. (A) Map view of the Yingen basin, which is made up of many individual sub-basins and associated with metamorphic core complexes. (B) Map view of Early Cretaceous Songliao basin. Note the individualities of sub-basins. (C) A geologic section across the Yingen basin (a-a’ profile in A), showing marked synrift subsidence and minor postrift subsidence. (D) A geologic section across the southern Songliao basin (b-b’ profile in B) that is typified by pronounced postrift subsidence. Abbreviations: K_1_ = Lower Cretaceous, K_1y_ = Yixian Formation, K_1j_ = Jiufotang Formation, K_1s_ = Shahai Formation, K_1f_ = Fuxin Formation.

The relative timing of extension and volcanism is pivotal in discriminating different types of rift basins, which can be readily recognized by their distinct stratigraphic sequences (Fig. 4). Passive rift basins develop when the lithosphere is stretched and thinned, with the asthenosphere rising passively (Fig. 4A-a). Magma is then generated either by melting of the crust and lithospheric mantle due to asthenospheric heating or by decompressional melting of the asthenosphere. As a result, volcanic eruptions take place in the late stage of rifting when the lithosphere is considerably attenuated (Fig. 4A-b). Passive rift basin development is thus recorded by basin sequences typified by the lower clastic rocks and the upper volcanic/volcaniclastic rocks (Fig. 4A-c). By contrast, extensive volcanism usually precedes subsidence of active rift basins as a consequence of the active asthenospheric upwelling and crustal doming (Fig. 4B-a). The domed upper crust then experiences horizontal stretching owing to gravitational instability and collapse, thereby forming active rift basins in the extended areas. Clastic sedimentation is therefore characteristic of the late stage of active rift basins (Fig. 4B-b). Typical volcano-sedimentary sequences of active rift basins are accordingly marked by a lower volcanic part and an upper clastic part (Fig. 4B-c). As regards wide rift basins, they initiate and develop owing to gravitational collapse of the thickened orogenic crust, as manifested by broad occurrence of small-scale disparate rift basins in the upper crust (Fig. 4C-a). The isolated basins expand through lateral linkage of adjacent basins, and thus often express themselves as elongated or narrow basins in map view (Fig. 5). Continued gravitational spreading can result in stress localization and may eventually give rise to metamorphic core complexes (MCCs) [99]. The close association of MCCs with wide rifting can be exemplified by the presence of a number of Early Cretaceous MCCs, like the Yagan MCC [29,106], Ereendavaa MCC [107] and Ulan-Ude MCC [33] in the ECAO. Magmatism also occurs simultaneously with wide rifting (Fig. 4C-b) and is well documented [108–110]. Potential heat sources for partial melting of thickened crust might be internal heat production by radioactive decay [111] and/or heat flux related to the asthenospheric upwelling triggered possibly by plate subduction [112]. Widespread volcanism in the ECAO is commonly attributed to subduction-induced delamination [69]. Wide rifts are marked by basin sequences dominated by clastic facies, with volcanic interlayers being present at different stratigraphic levels (Fig. 4C-c).

Early Cretaceous rift basins in NE Asia are categorized into three types in this study: passive, active and wide rift basins. Rift basins in the eastern NCC display similar synrift stratigraphic successions that begin with clastic units characterized by alluvia/fluvial and lacustrine facies associations. The clastic units are overlain by upper units dominated by volcanic and volcaniclastic rocks. These typical synrift sequences are well manifested in the Jiaolai and Hefei basins (Fig. 2). The Laiyang Group and Zhuxiang Formation represent the lower clastic units, while the Qingshan Group and Maotanchang Formation exemplify the upper volcanic units (Figs 2 and 4A-d). Rift basins in the eastern NCC thus fall into passive rift basins. Rift basins in the northern NCC show synrift sequences typified by a lower volcanic unit and an upper clastic unit, contrasting strikingly with rift basin sequences in the eastern NCC. The lower unit is represented by the Donglingtai, Zhangjiakou and Yixian volcanics, while the Dabeigou, Xiguayuan and Jiufotang

Formations make up the upper clastic units in the Luanping and Beipiao basins, respectively (Figs 2 and 4B-d). The rift basins in the northern NCC are therefore considered as active rift basins. Early Cretaceous basins in the ECAO have been well investigated and classified as wide rift basins [29,37,80]. The extensive distribution of small-scale basins, as manifested by Early Cretaceous basin families in the Yingen and Songliao basins (Fig. 5), typifies the wide rift basins. Volcanic layers of various thicknesses occur at different levels of successions of the wide rift basins (Figs 2 and 4C-d), as displayed by Lower Cretaceous sequences in the Songliao basin [21,71,72], the Great Xing’an Range [68], the Hailar basin [66,67] and the Erlian basin [66].

It is noteworthy that the distinct types of rift basins occurred in different regions in NE Asia during the Early Cretaceous: wide rift basins developed in the ECAO, active rift basins in the northern NCC and passive rift basins in the eastern NCC (Fig. 1). The wide rift basins are reminiscent of the Tertiary Basin and Range Province of the United States [113,114], and were attributed to gravitationally driven collapse of thickened and heated orogenic crust [29,37,115,116]. Other drivers are also proposed for the Early Cretaceous extension, such as backarc crustal extension [21,117] or transtension in association with escape tectonics [9,118]. However, these mechanisms can hardly explain the extensive distribution of these supracrustal basin families. Passive rift basins in the eastern NCC resulted from backarc extension triggered by high-angle subduction of the paleo-Pacific plate [18,119], with regional tensional stress oriented NW to SE [120,121]. The Tanlu fault behaved as a major normal fault in the Early Cretaceous, playing a major role in subsidence of the Hefei, Jiaolai and other adjacent basins [25]. It has been bewildering how an active rift basin was induced in the northern NCC.

The Late Cretaceous saw a period when most rift basins experienced vertical motion in NE Asia [21,29]. Basin subsidence and sedimentation in eastern NCC were partly associated with normal faulting [25,26]. Magmatism became quiet, and was only active at the eastern edge of the NE Asian continent, such as the Sikhote Alin belt, southeast Korea and southwest Japan [3,4,91]. The passive rift basins in the eastern NCC subsided as a result of N–S extension, as exemplified by the Jiaolai basin where the Upper Cretaceous Wangshi Group was deposited under the control of E–W-striking normal faults like the Baichihe and Pingdu faults [26]. The N–S extension was postulated to have resulted from transcurrent tectonics [5]. E–W-trending normal faulting also took place in the Hefei basins, and controlled sedimentation of the Zhangqiao Formation [25]. Contraction happened in the easternmost NE Asian continent at the end of the Early Cretaceous, as registered by a regional unconformity beneath Upper Cretaceous strata in a number of basins, such as the Hefei, Jiaolai, Songliao and Sanjiang basins [21,26,122]. Strong transpression occurred along strike-slip fault zones, leading to folding and uplifting of Lower Cretaceous successions of the basins near or within the fault zones, as indicated by intense deformation of Lower Cretaceous strata in the Sanjiang basin [122] and the Yisu basin [26]. The basins far away from the strike-slip faults only experienced a short-lived vertical uplift, with no obvious break in the Lower–Upper Cretaceous successions. For instance, Lower and Upper Cretaceous strata are conformable in the Gyeongsang basin in SE Korea [52], and the Cretaceous synrift and postrift sequence is only separated by a short-termed disconformity in the Songliao basin [21].

Most rift basins in the ECAO underwent minor subsidence in the postrift stage, with postrift successions usually <800 m thick [29]. The insignificant postrift subsidence resulted possibly from lower-crustal flows from the less stretched areas to the strongly attenuated regions [29]. The lower-crustal flows prevented the crust of the rift basins from further thinning, thereby reducing postrift tectonic subsidence. The Songliao basin is an exception in that it underwent striking postrift subsidence with sedimentary successions up to 5000 m thick [21]. Opinions diverge on the origins of large-magnitude postrift subsidence of the Songliao basin. It is assumed that the lithosphere of the Songliao basin was significantly thinned due to backarc extension, and subsequent thermal contraction of the asthenosphere was thus responsible for the pronounced postrift subsidence [21]. Li and Liu attributed the marked postrift subsidence to the superposition of dynamic subsidence induced by downward dragging of the subducting paleo-Pacific plate [123]. It is also argued that west-verging thrusting on the eastern margin of the Songliao basin might have contributed partially to the postrift subsidence, albeit thermal subsidence was dominant in the early stage [122]. Transpressional deformation was localized along the strike-slip fault zones in the easternmost margin of NE China at the Early to Late Cretaceous boundary, like the Yilan–Yitong and Dunhua–Mishan faults [5,40], and led to inversion of the Sanjiang basins in between [122]. The Songliao basin did not undergo shortening until ∼80 Ma when all the basin's fills were folded to various degrees and partially uplifted under roughly west–east compression [21].

Upper Cretaceous strata are considerably thin and only occur in a few rift basins in the northern NCC (Fig. 2). It is unclear why the active rift basins largely came to an end in the Late Cretaceous. Most Early Cretaceous successions remain fairly flat, indicating weak deformation. It is plausible that the active rift basins terminated as a result of vertical crustal motion rather than horizontal shortening. The northern NCC in practice experienced polyphase rapid uplifting in the Cretaceous, starting ∼120 Ma based on low-temperature thermochronologic data [124]. The episodic vertical motion might be responsible for the lack of Upper Cretaceous strata. More work is obviously needed to explore the driver of the polyphase uplift/denudation of the northern NCC. The end-Early Cretaceous thrusting was rarely recorded in the northern NCC except in a few localities where sinistral transpressive faults, such as the Nantianmen and Yaowangmiao faults in western Liaoning, displace the Mesoproterozoic dolostones over Lower Cretaceous strata [125].

## DYNAMICS OF CRETACEOUS BASINS

**Figure 6.**
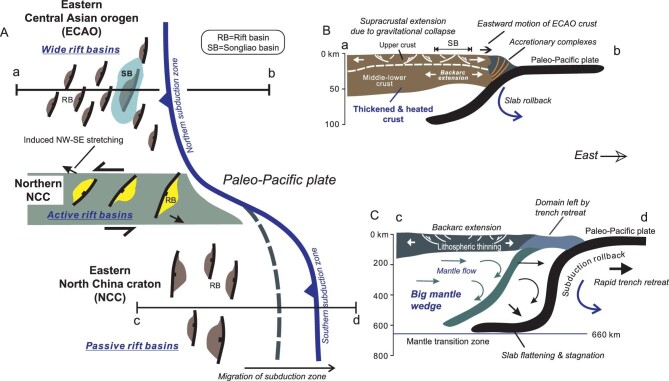
Diagram showing a possible linkage between Early Cretaceous basins and the paleo-Pacific plate subduction. (A) Passive rift basins occur in the NCC, bounded on the east by the southern subduction zone. Wide rift basins develop in the ECAO, bounded by the northern subduction zone. Active rift basins happen in the northern NCC. (B) A sketch showing that wide rift basins result from supracrustal stretching due to gravitational collapse of the thickened ECAO crust. The Songliao basin crust is significantly thinned owing to the superposition of backarc extension induced by paleo-Pacific plate subduction. Note that the northern subduction zone was relatively fixed in Jurassic to Early Cretaceous times. (C) A sketch showing that passive rift basins originate from backarc extension triggered by a combination of rollback and retreat of the subducting paleo-Pacific plate. A big mantle wedge might have begun developing beneath the NCC since the Early Cretaceous.

Distinct types of rift basins developed in different regions in NE Asia during the Early Cretaceous, and there should be a coherent mechanism that governed spatial distribution of the diverse rift basins. We here advance a tectonic model that seems to better explain why different rifting took place in different regions in the Early Cretaceous (Fig. 6). The NE Asian continent was bounded on the east by a subduction zone, which presumably initiated in the Early Jurassic, as implied by the presence of Early Jurassic accretionary complexes and arc/backarc igneous associations at the eastern margin of the ECAO [4,14,126]. Early Cretaceous arc volcanic rocks are rarely documented in the eastern edge of the NCC, but mafic and felsic intrusives in the eastern NCC implicate active subduction of the paleo-Pacific plate [87]. One possibility is that the subduction zone was far away from the present-day eastern edge of the NCC continent, and the Early Cretaceous island-arc belt might have been destroyed by later subduction and/or transform processes due to reorganization of the western Pacific plate [127,128]. This situation hints at a change in the paleo-Pacific plate subduction process along the subduction zone (Fig. 6A). The whole subduction zone could be divided into the northern and southern segments (Fig. 6). The northern subduction zone east of the ECAO was relatively fixed, as evidenced by complete preservation of Jurassic–Early Cretaceous accretionary complexes in the Nadanhada and Sikhote Alin belts [41,126]. In contrast, a lack of geologic records of Jurassic–Early Cretaceous arc systems suggests that the subduction zone east of the NCC might have been located far away from the continental margin. It is thus plausible that a transfer zone developed to accommodate the different subduction processes at the northern and southern subduction zones. The transfer zone just occurred beneath the northern NCC (Fig. 6A).

An internal connection might exist between the paleo-Pacific plate subduction and extensional tectonics in the ECAO during the Early Cretaceous (Fig. 6B). The paleo-Pacific plate subduction not only produced an accretionary prism, as evidenced by the Late Jurassic–Early Cretaceous Nadanhada and Sikhote-Alin complexes [41,126], but also induced backarc extension, as implicated by Early Cretaceous bimodal volcanism and A-type rhyolite [4,20]. The free eastern boundary could have facilitated extensional collapse of the thickened ECAO crust, leading to the formation of wide rift basins. The superposition of backarc extension and gravitational collapse brought about significant thinning of the whole lithosphere in the eastern ECAO (Fig. 6B), thereby resulting in uprising of the asthenosphere and voluminous volcanic eruption in both the Songliao basin and Great Xing’an Range [20,69,81]. This mechanism offers a satisfactory explanation for intense volcanism in the Great Xing’an Range in the Early Cretaceous and the pronounced thermal subsidence of the Songliao basin in the Late Cretaceous.

It was argued that Early Cretaceous magmatism was younging to the east in the ECAO [69]. Close scrutiny of available geochronologic data, however, shows that magmatism occurred throughout the ECAO mainly in the timespan from 135 to 110 Ma [32,69,81,97,110] and did not display the marked eastward progression. Extensive volcanism across the ECAO is attributed either to the break-off of the subducting plate [20] or delamination of subcontinental lithosphere [23]. Although the two tectonic models could explain extensive magmatic outpouring and uplifting of wide rift systems in the late Early Cretaceous, they can hardly account for why significant thermal subsidence only happened in the Songliao basin. We tentatively ascribe Early Cretaceous magmatism in the ECAO interior to the combination of internal heating due to radioactive decay in the thickened crust and heat flux of the asthenospheric upwelling triggered by plate subduction.

Passive rifting in the eastern NCC resulted from horizontal lithospheric extension, which was presumably induced by rollback and retreat of the subducting paleo-Pacific plate [10,25]. The westward decrease in the intensity of crustal stretching implies that horizontal tensile force must have been applied from the east. Given that there are no records of Early Cretaceous arc magmatism in the present-day eastern edge of the NCC, the subduction zone must have been located far to the east during the Early Cretaceous. It is conjectured that the southern subduction zone had continued migrating eastward with time owing to persistent rollback and/or retreat of the subducting paleo-Pacific plate (Fig. 6C). Continued rollback and/or retreat of paleo-Pacific plate subduction might also have led to the formation of a big mantle wedge beneath the NCC in the Early Cretaceous, which in turn promoted lithospheric thinning of the eastern NCC by means of water-assisted thermal erosion [10].

Two-dimensional thermal mechanical modeling was recently performed to investigate behaviors of the overriding continent with differing thermal states in the process of oceanic plate subduction [129]. It is shown that: (i) trenchward thrusting of overthickened and hot (>17.5°C km^−1^) crust will slow down the trench retreat; and (ii) decoupling could occur between the overriding continents and subducting oceanic plates if continents possess low thermal gradients (∼10–15°C km^−1^) and normal crustal thickness. The modeling results carry important implications for subduction processes of the western paleo-Pacific plate. As discussed earlier, complete Jurassic–Cretaceous accretionary complexes are well preserved at the eastern margin of the ECAO. This fact implicates that the northern subduction zone must have been

relatively fixed or experienced little eastward retreat during the late Mesozoic, compatible with the prediction of the modeling [129]. In contrast, the NCC was a domain with relatively normal geotherm and crustal thickness as a whole. Given that few geologic records of arc systems have been identified along the eastern margin of the NCC, the southern subduction zone is thus inferred to have undergone eastward migration as a result of continuous trench retreat and subduction rollback of the paleo-Pacific plate. Both geologic observations and interpretations seem consistent with the numerical modeling [129].

**Figure 7.**
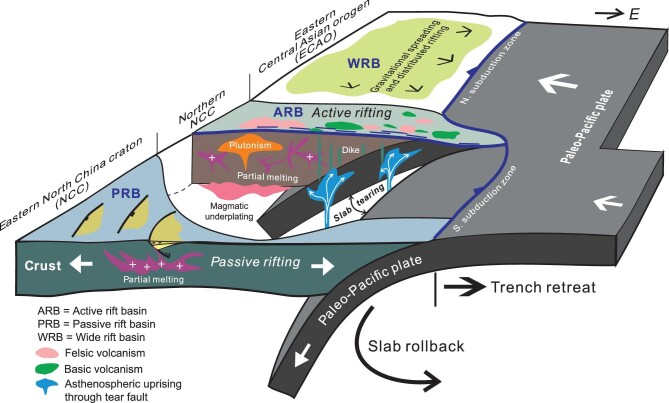
A tectonic model showing that slab tearing played an important role in controlling active rift basin evolution and high-flux magmatism in the northern NCC in the Early Cretaceous. Slab tearing possibly resulted from a higher rate of rollback and retreat of the subducting paleo-Pacific plate at the southern subduction zone. Hot mantle materials ascended through the tear fault and impinged on the overlying lithosphere, thus triggering magmatism and eastward-younging polarity as slab tearing progressed from west to east. Passive rift basins in the eastern NCC were generated by horizontal extension induced by rapid trench retreat in conjunction with subduction rollback. Wide rift basins developed due to gravitational collapse of the thickened crust of the ECAO and the northern subduction zone underwent not very much eastward migration.

Tearing of the subducting lithospheric slab has been widely documented by geophysical observations in many subduction zones around the world [130,131], and is attributed to the variation in rates of subduction rollback and trench retreat along the length of subduction zones [132]. We conjecture that the subducting paleo-Pacific plate experienced vertical slab tearing beneath the northern NCC as a result of different rollback and/or retreat velocities of the northern and southern subduction zones (Fig. 7). The northern subduction zone was relatively fixed between the Late Jurassic to Early Cretaceous, whereas the southern subduction zone continued migrating to the east, with only the backarc system left in the eastern NCC (Fig. 6A). Persistent eastward retreat of the southern subduction zone eventually led to segmentation of the subduction zone and the formation of a lithospheric-scale tear fault that split the subducting paleo-Pacific plate beneath the NE Asian continent (Fig. 7). One of the direct consequences is the ascent of the hot asthenospheric materials along the tear fault, which heated the overlying lithosphere and triggered vigorous magmatism in the northern NCC (Fig. 6). The plausibility of tearing of the subducting paleo-Pacific plate is sustained by several geologic facts. First, Early Cretaceous volcanism and plutonism occurred mostly in the northern NCC, as indicated by linear distribution of igneous rocks (Fig. 3). Second, Early Cretaceous volcanism showed an eastward younging polarity, taking place first in the west and shifting to the east [10,11] (Fig. 3). Third, Early Cretaceous igneous rocks in the northern NCC were formed at high temperatures and sourced partially from depleted mantle materials [11]. All the geologic records are compatible with the proposed slab tearing process. Tearing of the subducted

paleo-Pacific plate happened first in the west, thus permitting asthenospheric materials to penetrate through the slab gap produced by lithospheric-scale tearing (Fig. 7). Consequently, vigorous magmatism occurred first in the western segment of the northern NCC as a result of heating of the asthenospheric uprising, giving rise to the voluminous Donglingtai and Zhangjiakou volcanics. Tearing then propagated eastward and upward over time, and brought about eastward migration of magmatic activities (Fig. 7). Slab tearing thus offers a good explanation for the generation of voluminous volcanism and active rift basins in the northern NCC (Fig. 7).

Also noticeable is the occurrence of a number of Early Cretaceous MCCs in the northern NCC (Fig. 1), such as the Hohhot [133], Yunmengshan [134,135], Yiwulushan [136] and Liaonan MCCs [137], indicating that the northern NCC was a highly extended corridor. The MCCs possess two important aspects: (i) footwalls or the lower plates usually contain Early Cretaceous plutons; and (ii) detachments experience high temperature (up to 600°C) ductile shearing [109]. The facts imply that magmatism must have played an important role in the MCCs’ formation by thermally weakening the lithosphere/crust. MCCs preferentially form in the hot and thickened crust, as revealed by both natural examples and numerical modeling [99,100,138]. The northern NCC had experienced two strong shortening events prior to the Early Cretaceous [2,15,16], and had already evolved into an intraplate orogen with the considerably thickened crust. Subsequent voluminous magmatic activity must have further weakened the northern NCC [109]. Therefore, the northern NCC behaved as a unique extensional corridor affected by both magmatic uprising and gravitational collapsing in the Early Cretaceous, and was thus prone to active rifting and MCC formation.

A global plate reorganization event happened at ∼105–90 Ma, and the paleo-Pacific plate began moving to the north or north-northwest [127]. The N- or NNW-directed movement of the paleo-Pacific plate strongly sheared the eastern margin of the NE Asian continent from the beginning of the Late Cretaceous [41,127,139], contrasting with the dominant NW-directed subduction in the Early Cretaceous [25]. The kinematic change in direction, rate and subduction angle of the paleo-Pacific plate led to two prominent consequences in the eastern margin of NE Asia in the Late Cretaceous time: (i) reactivation and/or initiation of a number of large-scale left-slip faults, such as the Tanlu fault [5], Dunhua-Mishan fault [40], Median tectonic lines [140], South Korea tectonic line [141] and Central Sikhote Alin fault [41]; (ii) transpressional deformation in the period from 97 to 80 Ma, which presumably resulted from an abrupt increase in subduction rate of the paleo-Pacific plate [139]. The transgression resulted in inversion of Early Cretaceous rift basins in the eastern margin of the NE Asian continent, such as the Hefei, Jiaolai, Yisu, Sanjiang and Songliao basins [21,26,122]. Following the shortening event, rifting resumed as a consequence of N-S extension in some localities, and possibly bore upon persistent large-scale left-slip faulting [26,120,141]. Late Cretaceous magmatism was thus concentrated along the easternmost margin of the NE Asian continent [41,96], and became significantly weak toward the interior due to localization of transcurrent deformation [3,5]. The Songliao basin underwent marked postrift subsidence in the Late Cretaceous tectonic quiescence [21]. Most of the NE Asia continent experienced vertical uplift or minor subsidence in the Late Cretaceous on account of the scarcity of Upper Cretaceous strata [122].

Admittedly, uncertainties and disagreements remain regarding the subduction history of the western paleo-Pacific plate in the late Mesozoic. The existing reconstructions of paleo-Pacific plate subduction need to be refined when new data are available. A more feasible mechanism for tectonic development of Cretaceous basins in NE Asia awaits a better understanding of subduction processes and the kinematic history of the paleo-Pacific plate in the late Mesozoic.

## CONCLUSION

Cretaceous rift basins characterize the NE Asian continent. Three types of rift basin are identified according to their distinct volcano-sedimentary sequences and subsidence history, and termed as passive, active and wide rift basins. Passive rift basins in the eastern NCC commenced with clastic deposition, which was followed by volcanic eruption. Active rift basins were formed in the northern NCC and marked by vigorous volcanism at the beginning of basin history. Clastic sedimentation then took place and became more prevalent with time. Wide rift basins occurred in the ECAO and were mostly filled with clastics. Volcanic and volcaniclastic layers were present throughout basin successions, and abundant in the eastern ECAO. The passive rift basins are attributed to horizontal lithospheric stretching induced by rollback and retreat of the subducting paleo-Pacific plate. The wide rift basins originate from gravitational collapse of the hot and thickened crust. Development of active rift basins is presumably related to asthenospheric uprising through a lithospheric-scale tear fault. The Late Cretaceous was a period of tectonic quiescence, and most of the Early Cretaceous rift basins experienced either sagging or uplift. Late Cretaceous crustal deformation was localized along the eastern margin of the NE Asian continent in response to kinematic change of the paleo-Pacific plate that began moving to the north or north-northwest. Basins near or within major strike-slip faults in the eastern margin were either inversed or developed into strike-slip basins.
